# Chapter F of the *International Code of Nomenclature for algae, fungi, and plants* as approved by the 11th International Mycological Congress, San Juan, Puerto Rico, July 2018

**DOI:** 10.1186/s43008-019-0019-1

**Published:** 2019-12-27

**Authors:** Tom W. May, Scott A. Redhead, Konstanze Bensch, David L. Hawksworth, James Lendemer, Lorenzo Lombard, Nicholas J. Turland

**Affiliations:** 1 Royal Botanic Gardens Victoria, Birdwood Avenue, Melbourne, VIC 3004 Australia; 2 Ottawa Research and Development Centre, Science and Technology Branch, Agriculture and Agri-Food Canada, 960 Carling Avenue, K.W. Neatby Building, Ottawa, ON K1A 0C6 Canada; 3 Botanische Staatssammlung München, Menzinger Str. 67, 80638 Munich, Germany; 4 Westerdijk Fungal Biodiversity Institute, Uppsalalaan 8, Utrecht, CT 3584 The Netherlands; 5 Comparative Plant and Fungal Biology, Royal Botanic Gardens, Kew, Surrey TW9 3DS UK; 6 Department of Life Sciences, The Natural History Museum, Cromwell Road, London, SW7 5BD UK; 7 Jilin Agricultural University, Changchun, 130118 Jilin Province China; 8 Institute of Systematic Botany, New York Botanical Garden, 2900 Southern Boulevard, Bronx, NY 10458-5126 USA; 9 Botanischer Garten und Botanisches Museum Berlin, Freie Universität Berlin, Königin-Luise-Str. 6–8, 14195 Berlin, Germany

**Keywords:** Editorial Committee for Fungi, Governance, Identifiers, Repositories, Sanctioning, *San Juan Chapter F*, *Shenzhen Code*, Taxonomy

## Abstract

A revised version of *Chapter F* of the *International Code of Nomenclature for algae, fungi, and plants* is presented, incorporating amendments approved by the Fungal Nomenclature Session of the 11th International Mycological Congress held in San Juan, Puerto Rico in July 2018. The process leading to the amendments is outlined. Key changes in the *San Juan Chapter F* are (1) removal of option to use a colon to indicate the sanctioned status of a name, (2) introduction of correctability for incorrectly cited identifiers of names and typifications, and (3) introduction of option to use name identifiers in place of author citations. Examples have been added to aid the interpretation of new Articles and Recommendations, and Examples have also been added to the existing Art. F.3.7 concerning the protection extended to new combinations based on sanctioned names or basionyms of sanctioned names (which has been re-worded), and to Art. F.3.9 concerning typification of names accepted in the sanctioning works.

## INTRODUCTION

*Chapter F* of the *International Code of Nomenclature for algae, fungi, and plants* (*Code*) is the section of the *Code* that brings together provisions that deal solely with names of organisms treated as fungi. *Chapter F* was an innovation in the *Shenzhen Code* (Turland et al. 2018). Furthermore, Division III of the *Shenzhen Code,* the Provisions for Governance, included new procedures for amending *Chapter F*, so that proposals relating to the content of *Chapter F* are decided on by the Fungal Nomenclature Session (FNS) of an International Mycological Congress (IMC) (Hawksworth et al. 2017). The FNS of the 11th IMC was held on 19 July 2018. The revised version of *Chapter F* resulting from proposals accepted at this FNS is presented here, referred to as the *San Juan Chapter F*. The online version of the *Shenzhen Code* will be amended so that its *Chapter F* becomes the *San Juan Chapter F*, with new and edited material marked as such.

The *San Juan Chapter F* supersedes *Chapter F* of the *Shenzhen Code*. The rules of the *San Juan Chapter F* became effective immediately upon acceptance of the resolution at the closing plenary session of the 11th International Mycological Congress (IMC11) on 21 July 2018, that the decisions and appointments of its Fungal Nomenclature Session be approved. Previous *Codes* have been translated into various languages. If the *San Juan Chapter F* is translated into other languages, the English version will be definitive in questions about the meaning of provisions in any translations.

## AMENDING CHAPTER F

Seven formal proposals to amend *Chapter F* of the *Shenzhen Code* were published in *IMA Fungus*, the journal of the International Mycological Association, on 1 April 2018 (Hawksworth 2018). A synopsis of these proposals, with comments by the Secretary (T.W. May) and Deputy Secretary (S.A. Redhead) of the Fungal Nomenclature Bureau, was published in *IMA Fungus* on 23 May 2018 (May and Redhead 2018). This synopsis served as the basis for the preliminary guiding vote that was held online, opening on 22 May 2018 and closing on 17 June 2018. As specified by Division III of the *Shenzhen Code*, participation in the guiding vote was extended to (1) authors of proposals, (2) members of the Nomenclature Committee for Fungi (NCF), (3) members of the International Mycological Association (IMA), and of its Member Mycological Organizations (MMOs), and (4) members of four additional organizations that were nominated by the Fungal Nomenclature Bureau. Results of the guiding vote were published in *IMA Fungus* on 13 July 2018 (May and Miller 2018). That report also included two further proposals, and in total 11 “proposals from the floor” were received prior to the commencement of the Fungal Nomenclature Session.

The Fungal Nomenclature Session met on 19 July 2018 in Room 201, Puerto Rico Convention Center, San Juan, Puerto Rico, during the main part of IMC11 held at the same Congress Centre from 16 to 21 July 2018. Any person registered for the day of the Congress on which the FNS was held was entitled to attend and vote; 149 such persons attended the FNS. Officers of the FNS, appointed in conformity with Division III of the *Shenzhen Code*, were Amy Rossman (Corvallis, USA, Chair), Tom W. May (Melbourne, Australia, Secretary), Scott A. Redhead (Ottawa, Canada, Deputy Secretary), Lorenzo Lombard (Utrecht, The Netherlands, Recorder), and five Deputy Chairs, David L. Hawksworth (London, UK, Deputy Chair Emeritus), Meredith Blackwell (Columbia, USA), Pedro Crous (Utrecht, The Netherlands), Karen Hughes (Knoxville, USA), and Yu Li (Changchun, China; in absentia). In addition, Nicholas J. Turland (Berlin, Germany), Rapporteur-général for the 2023 International Botanical Congress (IBC), attended as a non-voting advisor to the FNS, on the invitation of the International Mycological Association.

Procedures in the lead up to and during the FNS closely followed those of the Nomenclature Section of an IBC, except that during the FNS there were no “institutional” votes. Discussions of the FNS were held in English, and were recorded. The full proceedings of the San Juan FNS will form a separate publication, planned for early 2020, following the tradition of providing transcriptions of the proceedings of Nomenclature Sections of IBCs (e.g. Flann et al. 2014 for the Melbourne IBC).

An Editorial Committee for each *Code* is elected by the Nomenclature Section of the corresponding IBC. For *Chapter F*, it was desirable to mirror this process, and a proposal to amend Division III to make specific reference to an Editorial Committee for Fungi was put forward at the San Juan FNS. However, at the FNS this proposal was considered to be outside of the mandate of the FNS (see below). Instead, an ad hoc Editorial Committee for Fungi, as allowed under Div. III Prov. 5.2(e) and 8.1, was proposed and approved by the FNS at the beginning of the session prior to voting on individual proposals. Composition of the Editorial Committee for Fungi is Tom W. May (Melbourne, Australia, Secretary Fungal Nomenclature Bureau), Scott A. Redhead (Ottawa, Canada, Deputy Secretary Fungal Nomenclature Bureau), Konstanze Bensch (The Netherlands/Germany), David Hawksworth (London, UK, Deputy Chair Emeritus Fungal Nomenclature Bureau), James C. Lendemer (New York, USA), Lorenzo Lombard (Utrecht, The Netherlands), and Nicholas J. Turland (Berlin, Germany, Rapporteur-général of the 2023 IBC, Chair Editorial Committee). Membership is as approved by the FNS with addition of Lombard and Lendemer, who were elected by the Editorial Committee for Fungi after the Congress.

According to the Preface of the *Shenzhen Code*, the Editorial Committee has a mandate to “deal with matters specifically referred to it, to incorporate into the new *Code* the changes agreed by the Section, to clarify any ambiguous wording so long as the meaning is not changed, to ensure consistency and optimal placement of provisions while retaining the present numbering insofar as possible, and to add (or remove) Examples to best illustrate the provisions”. The Editorial Committee for Fungi proceeded as if with the same mandate; knowing that their work would be reviewed in due course by the Editorial Committee for the *Shenzhen Code*.

The “Report of Congress action on nomenclature proposals relating to fungi”, detailing the committees and officers appointed by the IMC and the results of the proposals was published in *IMA Fungus* on 9 November 2018 (May et al. 2018). This publication reported that, of the seven published proposals to amend *Chapter F* of the *Shenzhen Code,* two were accepted and two were referred to the Editorial Committee for Fungi; an additional proposal was accepted from among 11 new proposals made from the floor of the FNS. Five proposals (three introduced from the floor) were referred to two Special-purpose Committees, established by the FNS.

At the San Juan FNS, seven of the proposals from the floor related to governance, as covered in Division III. It was realized that these proposals were likely to be outside of the current mandate extended to the IMC, which is limited to “proposals relating to the content of Chapter F” (Div. III Prov. 8.1). Consequently, four of the seven proposals were withdrawn by their proposers and three were rejected. One such proposal concerned establishment of an “Editorial Committee for Fungi” (see above), the others related to the guiding vote. As discussed in the report on Congress actions (May et al. 2018) an opinion will be sought from the General Committee as to whether or not proposals to modify Division III (in relation to procedures restricted to mycological matters) can be dealt with at an IMC, or must be considered at an IBC.

A first draft of the *San Juan Chapter F*, incorporating the changes approved at IMC11, was prepared by TWM in October 2018, and distributed to the Editorial Committee for Fungi. After a number of rounds of discussion and editing, a draft was provided in late August 2019 to the Nomenclature Committee for Fungi and, as required by Div. III Prov. 8.10, to the Editorial Committee. Several weeks of intense discussion ensued, and a final draft was submitted to *IMA Fungus* on 9 September 2019.

We note that it was impractical for the Editorial Committee for Fungi to meet by teleconference due to the geographic spread of members (and consequent spread of time zones), and therefore all discussions were by e-mail. We recommend a face-to-face meeting of the Editorial Committee for Fungi as soon as possible after the FNS of the Amsterdam IMC in 2022, and consider that such a meeting would be beneficial in the expeditious preparation of a developed draft of the “*Amsterdam Chapter F*”.

The Editorial Committee for a given *Code* has in the past ceased active work once the relevant *Code* has been published. We note that members of the Editorial Committee were available and engaged positively at the final stage of revisions of the *San Juan Chapter F*. They will also need to be involved in four years hence, after the Amsterdam IMC, should any changes to the *San Juan Chapter F* be accepted at that Congress.

## AMENDMENTS TO CHAPTER F

Four new Articles and one new Recommendation have been inserted in the *San Juan Chapter F*, and a number of other Articles and Recommendations and a Note have been partially or substantially re-worded (Tables 1 and 2). New Examples have been provided in the new Articles and Recommendation, and Examples have also been added to a number of existing provisions. In comparison to *Chapter F* of the *Shenzhen Code*, existing provisions did not require re-numbering but some existing Examples required re-numbering due to insertion of new Examples. None of the new provisions is date-limited, and therefore they are retroactive (Principle VI). The new material is summarized at the head of the *San Juan Chapter F* (Appendix). We present here some further comments on the new material, and on two Articles that were the subject of an unsuccessful proposal, arranged in the order in which they appear in the *San Juan Chapter F*.

**Table 1:** List of changes to Articles, Notes, Recommendations and footnotes in the *San Juan Chapter F*

Numbering adopted in the *San Juan Chapter F* is used. No existing provisions needed re-numbering but some Examples were re-numbered.	
Art. F.3.2	Example added (Ex. 1)
Art. F.3.4	Example added (Ex. 5)
Art. F.3.7	reworded for clarity; Examples added (Ex. 8 and 9)
Art. F.3.9 and Note 2	Examples added (Ex. 10 and 11)
Rec. F.3A.1	significant revision; removal of colon as indicator of sanctioning (and Examples throughout *Chapter F* edited to conform)
Rec. F.3A.1 footnote	reworded to reflect revised version of Rec. F.3A.1
Art. F.5.1	Examples added (Ex. 2 and 3)
Art. F.5.2	new footnote; concerning identifiers for orthographical corrections
Art. F.5.4	minor revision; added “for the type designation” after identifier
Art. F.5 Note 4	minor revision; added “or guarantee” after constitute, to mirror Art. F.5 Note 1
Art. F.5.6–5.8	new; concerning incorrectly cited identifiers; Examples added (Ex. 4 and 5)
Rec. F.5A.1	some minor revisions and new clause *(c)*; authors should lodge electronic versions (such as PDFs) of publications with repositories
Rec. F.5A.2	minor revisions; deleted “accession”
Art. F.10	new; use of identifier in place of author citation
Rec. F.10A	as above; Example added (Ex. 1)

**Table 2:** Summary of changes incorporated in the *San Juan Chapter F*

In each case, the full wording of each Article, Note, Recommendation, Example and footnote should be consulted.
**• Art. F.3.7** reworded for clarity and Examples added concerning protection extended to names in sanctioning works used in new combinations.
**•** Selection of the type of a name adopted in one of the sanctioning works remains a choice among elements associated with the protologue and/or the sanctioning treatment [**Art. F.3.9**]. Wording unchanged, but Examples added.
**•** Sanctioning should be indicated only by “nom. sanct.” (the “:” option was removed) [**Rec. F.3A**].
**•** Repositories assign new identifiers for names with corrected orthography [**footnote** to **Art. F.5.2**].
**•** Errors in citation of identifiers are correctible, as long as the identifier was issued prior to publication of the nomenclatural novelty or typification [**Art. F.5.6**, **Art. F.5.8**].
**•** Subsequent validations of names associated with incorrectly cited identifiers are later isonyms and may be disregarded (as long as the identifier was issued for the isonym prior to its publication). Consequence of **Art F.5.6**.
**•** A new identifier must be obtained when validating a designation (an “invalid name”), even when that designation is associated with an existing identifier [**Art. F.5.7**].
**•** Authors are encouraged to provide electronic versions (such as PDFs) of publications containing nomenclatural novelties and typifications to the repository that issued the relevant identifiers [**Rec. F.5A.1**].
**•** Identifier issued for a name may be used in place of an author citation — but authors of a new name, a new combination, a name at a new rank, or a replacement name must still be cited in a protologue [**Art. F.10**, **Rec. F.10A**].

**Art. F.3.7** is conceded to be one of the more difficult Articles of the *Code* to comprehend on first acquaintance. There was no formal proposal to alter the Article, but the Editorial Committee for Fungi, in consultation with the Editorial Committee, revised the Article to make it more comprehensible. It would seem simplest to state that, within a given rank below genus, when sanctioned names are combined in new combinations protected status is retained over earlier non-sanctioned names (as long as the sanctioned names are available for the required combinations). However, “sanctioned names” are strictly names as they appear when accepted in the sanctioning works. Therefore, when a sanctioned name is a combination, its basionym is not itself sanctioned (despite use in the past of the term “sanctioned basionym”). Hence the device in Art. F.3.7 of referring to a name that “has the same type and final epithet as a sanctioned name” to cover names that are combinations based on sanctioned names or based on the basionyms of sanctioned names. Two Examples have been included to demonstrate the application of the Article.

There were two proposals to amend **Art. F.3.9**, concerning typification in relation to sanctioned names (Proposals *F-001* and *F-002*, proposed by Luis A. Parra and Juan C. Zamora in Hawksworth 2018). The intent of the proposals, in particular the first, was to allow neotypifications of sanctioned names to stand when such neotypifications were carried out despite elements from the context of the sanctioning work being available. The proposals were rejected, and Art. F.3 Note 2 referred to the Editorial Committee. After considering the Note, the Editorial Committee for Fungi decided that the current wording carried the intended meaning. Thus, where neotypifications have been undertaken in relation to types of names adopted in the sanctioning works, but there are elements associated with the context of the sanctioning work (such as cited illustrations or references to works that contain illustrations) that are suitable for selection as lectotypes, these neotypifications must be set aside (Art. 9.19), and lectotypification should be carried out. In this circumstance, if it is desired to maintain a particular usage, former neotypes may be selected as epitypes if they are not in conflict with the lectotype.

When it is appropriate to indicate the sanctioned status of a name, this is now recommended to be only by use of “nom. sanct.” (**Rec. F.3A**). The term “nom. sanct.” should be attached only to names accepted by sanctioning authors as they appear in the sanctioning works (i.e. “sanctioned names”). The term “nom. sanct.” should be attached neither to new combinations based on sanctioned names (where they are basionyms), nor to basionyms of sanctioned names (when the sanctioned name is a combination), nor to any combinations based on the basionym of a name that is sanctioned that are not themselves sanctioned. Use of “nom. sanct.” therefore parallels use of “nom. cons.”, which is attached to a name as conserved. Examples in the *San Juan Chapter F* that involve sanctioned names have all been edited in conformity with the new Rec. F.3A. The online version of the *Shenzhen Code* will reflect these changes in the *San Juan Chapter F*, but outside of *Chapter F* it retains the former indication of sanctioning (the abbreviation of the sanctioning author following a colon). After the next IBC, entries in the remainder of the *Code* that involve sanctioned names will be updated in conformity with the *San Juan Chapter F*, replacing the colon method by “nom. sanct.”.

Several new provisions were added in Art. F.5 concerning aspects of the registration of names and nomenclatural acts. **Art. F.5.6** and **Art. F.5.8** are new and provide for correctability of incorrectly cited identifiers. The new **Art. F.5.7** specifies that where a designation (an “invalid name”) has been introduced in association with an identifier, a new identifier must be obtained when validly publishing the former designation. Because Art. F.5.6 is not date-limited, it is retroactive (Principle VI), and consequently subsequent validations of names associated with incorrectly cited identifiers are later isonyms and may be disregarded (Art. 6 Note 2).

In relation to the practice of repositories recognized under Art. F.5.3, it is useful to distinguish the “issuing” of an identifier, in cases where a recognized repository issues an identifier to an author so that the author can comply with Art. F.5.1 or Art. F.5.4, from the “assigning” of an identifier, in situations where a repository assigns an identifier for internal database purposes, such as when adding to the repository a designation published without an identifier, or when making an orthographical correction. In the latter situation, the corrected version of the name is assigned a new identifier. The assigning of a new identifier to orthographical variants is not a *Code*-governed event, and is therefore not referred to under any Articles. However, a **footnote** has been added to Art. F.5.2 noting the practice of assigning new identifiers to names with corrected orthography.

**Rec. F.5A.1** was enlarged, with a new clause ***(c)***, to encourage authors of names to provide electronic versions of their publications to recognized repositories.

The new **Art. F.10** permits use of the identifier issued for a name by a recognized repository to be used in place of an author citation, subsequent to the valid publication of the name. The form specified is to cite the numerical portion of the identifier preceded by the # symbol, all enclosed in square brackets (**Rec. F.10A**). Nevertheless, when introducing names of new taxa, new combinations, names at new ranks, and replacement names conventional author citation is still to be used. It is important to note that while identifiers are issued by recognized repositories (currently three), data held by repositories, including identifiers, are shared on a regular basis. Therefore, when using an identifier in place of an author citation, the link may be to any repository that contains the required information.

## GLOSSARY AND APPENDICES

There are no modifications to the Glossary. The seven Appendices of the *Code* are currently available by querying an online database hosted by the Department of Botany at the Smithsonian National Museum of Natural History in Washington, DC, USA (https://naturalhistory2.si.edu/botany/codes-proposals/), maintained by John H. Wiersema. The Appendices consist of lists of conserved and rejected names, suppressed works and binding decisions that are added to as a result of: (1) consideration of proposals to conserve and reject, requests for binding decisions and requests to suppress works by Specialist Committees such as the Nomenclature Committee for Fungi (Div. III Prov. 7) in concert with the General Committee, and (2) consideration of lists of names for protection or rejection submitted under Art. F.2 or Art. F.7 (although no lists have yet been submitted under the latter provision). Final approval of material in the Appendices rests with an IBC, even when involving names of fungi or works exclusively devoted to fungi.

## FORMATTING AND STANDARDS

The *San Juan Chapter F* follows the formatting and standards used in the other sections of the *Code*. Further information on formatting and the citation of names of authors and bibliographic citations can be found in the Preface to the *Shenzhen Code* (pp. xxiii-xxiv). Recommendations and Notes are set in smaller type than the Articles, and the Examples and footnotes in smaller type than the Recommendations and Notes. These type sizes reflect the distinction between mandatory rules (Articles), complementary information or advice (Notes and Recommendations), and explanatory material (Examples and footnotes). Notes have binding effect but, unlike Articles, do not introduce any new provision or concept. Examples are distinguished, in addition to the smaller font size, by being indented.

## THE FUNGAL NOMENCLATURE SESSION AT IMC12 AND NEW PROPOSALS TO AMEND CHAPTER F

The *International Code of Nomenclature for algae, fungi, and plants* is amended by authority of the International Botanical Congress (IBC) except that its *Chapter F* is amended by authority of the International Mycological Congress (IMC); the *San Juan Chapter F* is the first such amendment. Provisions for the amendment of the *Code* can be found in its Division III, including specific provisions relating to amendments of *Chapter F*, which are dealt with at the Fungal Nomenclature Session of an IMC. The next International Mycological Congress, the twelfth (IMC12), will take place in Amsterdam, The Netherlands from 25 to 29 July 2022, with its Fungal Nomenclature Session held during this time. Proposals to amend the *San Juan Chapter F* may be published in *IMA Fungus* starting in 2020 and ending in early 2022. In early 2020, a notice will appear in *IMA Fungus* including an announcement that the journal will accept proposals and instructions on procedure and format. Proposals that are not submitted within the specified time frame cannot be included in the guiding vote. It is highly desirable that proposals are published well in advance of the IMC, to allow sufficient time for debate among the mycological community, and preparation of refinements or counter-proposals. Two Special-purpose Committees were established by the San Juan IMC: on “DNA sequences as Types for Fungi” and on “Names of Fungi with the Same Epithet”. These Committees will report to the Amsterdam IMC and are still in the process of being set up. Committee members will be appointed by the Nomenclature Committee for Fungi in consultation with the General Committee, and the membership will be announced in a report of the NCF, appearing in *IMA Fungus*.

## Data Availability

Not applicable.

## Appendix

**CHAPTER F**

**NAMES OF ORGANISMS TREATED AS FUNGI**

**(SAN JUAN VERSION)**

This Chapter brings together the provisions of this *Code* that deal solely with names of organisms treated as fungi.

Content in this Chapter may be modified by action of the Fungal Nomenclature Session of an International Mycological Congress (IMC) (see Div. III Prov. 8). The current version of this Chapter, the *San Juan Chapter F,* embodies the decisions accepted by the 11^th^ IMC in San Juan (Puerto Rico) on 21 July 2018.

**Always consult the online version of this** ***Code*** (http://www.iapt-taxon.org/nomen/main.php) in case of changes resulting from subsequent IMCs. The next IMC will be held in Amsterdam (The Netherlands) in 2022.

The following changes were introduced in the *San Juan Chapter F:*

Art. F.3.7. The Article was reworded to improve clarity, and two Examples were added.

Art. F.3.9. Two Examples were added.

Rec. F.3A. The option of using a colon to indicate sanctioning was removed. If it is desired to indicate sanctioning, it is recommended that this be done by using the abbreviation “nom. sanct.”.

Art. F.5. Several new provisions were added concerning aspects of the registration of names and nomenclatural acts. Art. F.5.6 allows correctability of incorrectly cited identifiers; Art. F.5.7 specifies that, in order for a designation that may be associated with an existing identifier to become a validly published name, a new identifier must be obtained; and Art. F.5.8 extends correctability to identifiers issued for type designations. Rec. F.5A.1 was enlarged to encourage authors of names to provide electronic versions of their publications to recognized repositories. A footnote was added to Art. F.5.2 noting the practice of assigning new identifiers to names with corrected orthography. Note that because Art. F.5.6 is not date-limited, it is retroactive (Principle VI), and consequently validations of names associated with incorrectly cited identifiers are later isonyms and may be disregarded (Art. 6 Note 2).

Art. F.10. A new Article was added concerning the use of identifiers in place of author citations.

Mycologists should note that the content of this *Code* outside of Chapter F pertains to all organisms covered by this *Code,* including fungi, unless expressly limited. This content includes rules about effective publication, valid publication, typification, legitimacy, and priority of names; citation and orthography; and names of hybrids.

Some provisions in the Preamble, Principles, Articles, and Recommendations elsewhere in this *Code,* such as those listed below, while not restricted to fungi, are of particular relevance to mycologists. **The full wording of these and all other relevant provisions of this** ***Code*** **should be consulted in all cases.**

Pre. 8. The provisions of this *Code* apply to all organisms traditionally treated as fungi, whether fossil or non-fossil, including chytrids, oomycetes, and slime moulds (but excluding *Microsporidia*).

Principle I. This *Code* applies to names of taxonomic groups treated as fungi, whether or not these groups were originally so treated.

Art. 4 Note 4. In classifying parasites, especially fungi, authors may distinguish within the species special forms (formae speciales) characterized by their adaptation to different hosts, but the nomenclature of special forms is not governed by the provisions of this *Code*.

Art. 8.4 (see also Art. 8 Ex. 12, Rec. 8B, Art. 40 Note 3, and Art. 40.8). Cultures of fungi are acceptable as types if preserved in a metabolically inactive state, and on or after 1 January 2019 this must be stated in the protologue.

Art. 14.15 and Art. 14 Note 4(c)(2). Before 1 January 1954, decisions on conservation of names made by the Special Committee for Fungi, became effective on 20 July 1950 at the VII International Botanical Congress in Stockholm.

Art. 16.3. Automatically typified suprafamilial names of fungi end as follows: division or phylum in *-mycota,* subdivision or subphylum in *-mycotina,* class in *-mycetes,* and subclass in *–mycetidae*. Automatically typified names not in accordance with these terminations are to be corrected.

Rec. 38E.1. The hosts should be indicated in descriptions or diagnoses of new taxa of parasitic organisms, especially fungi.

Art. 40.5. The type of a name of a new species or infraspecific taxon of non-fossil microfungi may be an effectively published illustration if there are technical difficulties of specimen preservation or if it is impossible to preserve a specimen that would show the features attributed to the taxon by the author of the name (but see Art. 40 Ex. 6, which treats representations of DNA sequences as falling outside of the definition of illustrations in Art. 6.1 footnote).

Art. 41.8(b) (see also Art. 41 Ex. 26). Failure to cite the place of valid publication of a basionym or replaced synonym, when explained by the backward shift of the starting date for some fungi, is a correctable error.

Art. 45.1 (see also Art. 45 Ex. 6 and 7 and Note 1). If a taxon originally assigned to a group not covered by this *Code* is treated as belonging to the algae or fungi, any of its names need satisfy only the requirements of the relevant other *Code* that the author was using for status equivalent to valid publication under this *Code*. Note especially that names of *Microsporidia* are not covered by this *Code* even when *Microsporidia* are considered as fungi.

**SECTION 1**

**LIMITATION OF THE PRINCIPLE OF PRIORITY**

**ARTICLE F.1**

**NOMENCLATURAL STARTING-POINT**

***F.1.1.*** Valid publication of names for non-fossil fungi (Pre. 8) is treated as beginning at 1 May 1753 (Linnaeus, *Species plantarum,* ed. 1, treated as having been published on that date; see Art. 13.1). For nomenclatural purposes, names given to lichens apply to their fungal component. Names of *Microsporidia* are governed by the *International Code of Zoological Nomenclature* (see Pre. 8).

***Note 1.*** For fossil fungi, see Art. 13.1(f).

**ARTICLE F.2**

**PROTECTED NAMES**

***F.2.1.*** In the interest of nomenclatural stability, for organisms treated as fungi, lists of names proposed for protection may be submitted to the General Committee, which will refer them to the Nomenclature Committee for Fungi (see Div. III Prov. 2.2, 7.9, and 7.10) for examination by subcommittees established by that Committee in consultation with the General Committee and appropriate international bodies. Protected names on these lists, which become part of the Appendices of the *Code* (see App. IIA, III, and IV) once reviewed and approved by the Nomenclature Committee for Fungi and the General Committee (see Art. 14.15 and Rec. 14A.1), are to be listed with their types and are treated as conserved against any competing listed or unlisted synonyms or homonyms (including sanctioned names), although conservation under Art. 14 overrides this protection. The lists of protected names remain open for revision through the procedures described in this Article (see also Art. F.7.1).

**ARTICLE F.3**

**SANCTIONED NAMES**

***F.3.1.*** Names in *Uredinales, Ustilaginales,* and *Gasteromycetes* (s. l.) adopted by Persoon (*Synopsis methodica fungorum,* 1801) and names of other fungi (excluding slime moulds) adopted by Fries (*Systema mycologicum,* vol. 1–3. 1821–1832, with additional *Index,* 1832; and *Elenchus fungorum,* vol. 1–2. 1828), are sanctioned.

***F.3.2.*** Names sanctioned are treated as if conserved against earlier homonyms and competing synonyms. Such names, once sanctioned, remain sanctioned even if elsewhere in the sanctioning works the sanctioning author does not recognize them. The spelling used when the name was sanctioned is treated as conserved, except for changes mandated by Art. 60 and F.9.

***Ex. 1.*** The name *Strigula smaragdula* Fr. (in Linnaea 5: 550. 1830) was accepted by Fries (Syst. Mycol., Index: 184. 1832) and therefore sanctioned. It is treated as if conserved against the competing earlier synonym *Phyllochoris elegans* Fée (Essai Crypt. Ecorc: xciv. 1825), which is the basionym of *Strigula elegans* (Fée) Müll. Arg. (in Linnaea 43: 41. 1880).

***Ex. 2.*** *Agaricus ericetorum* Pers. (Observ. Mycol. 1: 50. 1796) was accepted by Fries (Syst. Mycol. 1: 165. 1821), but later (Elench. Fung. 1: 22. 1828) regarded by him as a synonym of *A. umbelliferus* L. (Sp. Pl.: 1175. 1753), nom. sanct., and not included in his *Index* (p. 18. 1832) as an accepted name. Nevertheless *A. ericetorum* Pers. is a sanctioned name.

***Ex. 3.*** The spelling used when the name *Merulius lacrimans* (Wulfen) Schumach. was sanctioned (Fries, Syst. Mycol. 1: 328. 1821) is to be maintained, even though the epithet was spelled *‘lacrymans’* by Schumacher (Enum. Pl. 2: 371. 1803) and the basionym was originally published as *Boletus ‘lacrymans’* Wulfen (in Jacquin, Misc. Austriac. 2: 111. 1781).

***F.3.3.*** A sanctioned name is illegitimate if it is a later homonym of another sanctioned name (see also Art. 53).

***F.3.4.*** An earlier homonym of a sanctioned name is not made illegitimate by that sanctioning but is unavailable for use; if not otherwise illegitimate, it may serve as a basionym of another name or combination based on the same type (see also Art. 55.3).

***Ex. 4.*** *Patellaria* Hoffm. (Descr. Pl. Cl. Crypt. 1: 33, 54, 55. 1789) is an earlier homonym of the sanctioned generic name *Patellaria* Fr. (Syst. Mycol. 2: 158. 1822). Hoffmann’s name is legitimate but unavailable for use. *Lecanidion* Endl. (Fl. Poson.: 46. 1830), based on the same type as *Patellaria* Fr., nom. sanct., is illegitimate under Art. 52.1.

***Ex. 5.*** *Antennaria* Gaertn. (Fruct. Sem. Pl. 2: 410. 1791), in order to become available for use, required conservation against the later homonym *Antennaria* Link (in Neues J. Bot. 3(1,2): 16. 1809), nom. sanct. (Fries, Syst. Mycol. 1: xlvii. 1821).

***Ex. 6.*** *Agaricus cervinus* Schaeff. (Fung. Bavar. Palat. Nasc. 4: 6. 1774) is an earlier homonym of the sanctioned name *A. cervinus* Hoffm. (Nomencl. Fung. 1: t. 2, fig. 2. 1789), nom. sanct. (Fries, Syst. Mycol. 1: 82. 1821); Schaeffer’s name is unavailable for use, but it is legitimate and may serve as basionym for combinations in other genera. In *Pluteus* Fr. the combination is cited as *P. cervinus* (Schaeff.) P. Kumm. and has priority over the heterotypic (taxonomic) synonym *P. atricapillus* (Batsch) Fayod, based on *A. atricapillus* Batsch (Elench. Fung.: 77. 1786).

***F.3.5.*** When, for a taxon at a rank from family to genus, inclusive, two or more sanctioned names compete, Art. 11.3 governs the choice of the correct name (see also Art. F.3.7).

***F.3.6.*** When, for a taxon at a rank lower than genus, two or more sanctioned names and/or two or more names with the same final epithet and type as a sanctioned name compete, Art. 11.4 governs the choice of the correct name.

***Note 1.*** The date of sanctioning does not affect the date of valid publication, and therefore priority (Art. 11), of a sanctioned name. In particular, when two or more homonyms are sanctioned only the earliest of them may be used because the later one(s) are illegitimate under Art. F.3.3.

***Ex. 7.*** Fries (Syst. Mycol. 1: 41. 1821) accepted and thus sanctioned *Agaricus flavovirens* Pers. (in Hoffmann, Abbild. Schwämme 3: t. 24. 1793) and treated *A. equestris* L. (Sp. Pl.: 1173. 1753) as a synonym. He later (Elench. Fung. 1: 6. 1828) accepted *A. equestris,* stating “Nomen prius et aptius certe restituendum [The prior and more apt name is certainly to be restored]”. Both names are sanctioned, but, when they are treated as synonyms, *A. equestris* L*.*, nom. sanct. is to be used because it has priority.

***F.3.7.*** A name that neither is sanctioned nor has the same type and final epithet as a sanctioned name at the same rank may not be used for a taxon that includes the type of a sanctioned name at that rank unless the final epithet of the sanctioned name is not available for the required combination (see Art. 11.4(c)).

***Ex. 8*****.** The name *Agaricus involutus* Batsch (Elench. Fung.: 39. 1786) was sanctioned by Fries (Syst. Mycol. 1: 271. 1821) and therefore, when treated in *Paxillus* Fr. with the earlier but non-sanctioned name *A. contiguus* Bull. (Herb. Fr. 5: t. 240. 1785) as a synonym, the correct name is *P. involutus* (Batsch) Fr.

***Ex. 9*****.** The name *Polyporus brumalis* (Pers.) Fr. (Observ. Mycol. 2: 255. 1818), nom. sanct. (Fries, Syst. Mycol. 1: 348. 1821), based on *Boletus brumalis* Pers. (in Neues Mag. Bot. 1: 107. 1794), was treated by Zmitrovich & Kovalenko (in Int. J. Med. Mushr. 18: 23–38, suppl. 2: [2]. 2015) as synonymous with *B. hypocrateriformis* Schrank (Baier. Fl. 2: 621. 1789) and placed in *Lentinus* Fr., nom. sanct., in which the correct name is *L. brumalis* (Pers.) Zmitr. (in Int. J. Med. Mushr. 12: 88. 2010).

***F.3.8.*** Conservation (Art. 14), protection (Art. F.2), and explicit rejection (Art. 56 and F.7) override sanctioning.

***F.3.9.*** The type of a name of a species or infraspecific taxon adopted in one of the works specified in Art. F.3.1, and thereby sanctioned, may be selected from among the elements associated with the name in the protologue and/or the sanctioning treatment.

***Note 2.*** For names falling under Art. F.3.9, elements from the context of the protologue are original material and those from the context of the sanctioning work are considered as equivalent to original material.

***Ex. 10.*** When Stadler & al. (in IMA Fungus 5: 61. 2014) designated the lectotype of *Clavaria hypoxylon* L. (Sp. Pl.: 1182. 1753), sanctioned by Fries (Syst. Mycol. 2: 327. 1823) as *Sphaeria hypoxylon* (L.) Pers. (Observ. Mycol. 1: 20. 1796), they selected a specimen in K distributed by Fries (Scler. Suec. No. 181) and cited by him in the sanctioning treatment rather than any of the elements associated with the protologue.

***Ex. 11.*** In the absence of any specimens or illustrations from the context of the protologue that are original material, Peterson (in Amer. J. Bot. 63: 313. 1976) designated a specimen in L as the neotype of *Clavaria formosa* Pers. (Comm. Fung. Clav.: 41. 1797), nom. sanct. However, when sanctioning *C. formosa*, Fries (Syst. Mycol. 1: 466. 1821) cited several illustrations, which are therefore considered as equivalent to original material. Peterson’s neotypification was not therefore designated in conformity with Art. 9.13 and is not to be followed (Art. 9.19). Instead, Franchi & Marchetti (in Riv. Micol. 59: 323. 2017) designated as the lectotype of *C. formosa* one of the illustrations (Persoon, Icon. Desc. Fung. Min. Cognit. 1: t. III, fig. 6. 1798) that was cited by Fries (l.c., as “f. 5”).

***F.3.10.*** When a sanctioning author accepted an earlier name but did not include, even implicitly, any element associated with its protologue, or when the protologue did not include the subsequently designated type of the sanctioned name, the sanctioning author is considered to have created a later homonym, treated as if conserved (see also Art. 48).

***Note 3.*** For typification of sanctioned generic names, see Art. 10.2. Note that automatic typification under Art. 7.5 does not apply to sanctioned names. For legitimacy of sanctioned names (or names based on them), see also Art. 6.4, 52.1, 53.1, and 55.3.

Recommendation F.3A

***F.3A.1.*** When it is considered useful to indicate the nomenclatural status of a sanctioned name (Art. F.3.1), the abbreviation “nom. sanct.” (*nomen sanctionatum*) should be added in a formal citation; the place of sanctioning should also be added in full nomenclatural citations.^1^

***Ex. 1.*** *Boletus piperatus* Bull. (Herb. France: t. 451, fig. 2. 1790) was adopted in Fries (Syst. Mycol. 1: 388. 1821) and was thereby sanctioned. Depending on the level of nomenclatural information being presented, it should be cited as *B. piperatus* Bull., nom. sanct.; or *B. piperatus* Bull. 1790, nom. sanct.; or *B. piperatus* Bull., Herb. France: t. 451, fig. 2. 1790, nom. sanct.; or *B. piperatus* Bull., Herb. France: t. 451, fig. 2. 1790, nom. sanct. (Fries, Syst. Mycol. 1: 388. 1821).

***Ex. 2.*** *Agaricus compactus* [unranked] *sarcocephalus* (Fr.) Fr. was sanctioned when adopted by Fries (Syst. Mycol. 1: 290. 1821). That status should be indicated by citing it as *A. compactus* [unranked] *sarcocephalus* (Fr.) Fr., nom. sanct. The abbreviation “nom. sanct.” should not be added when citing its basionym *A. sarcocephalus* Fr. (Observ. Mycol. 1: 51. 1815) or when citing subsequent combinations such as *Psathyrella sarcocephala* (Fr.) Singer (in Lilloa 22: 468. 1949).

**SECTION 2**

**VALID PUBLICATION AND TYPIFICATION OF NAMES**

**ARTICLE F.4**

**MISPLACED RANK-DENOTING TERMS**

***F.4.1.*** A name is not validly published if it is given to a taxon of which the rank is at the same time, contrary to Art. 5, denoted by a misplaced term (Art. 37.6), but an exception is made for names of the subdivisions of genera termed tribes (tribus) in Fries’s *Systema mycologicum,* which are treated as validly published names of unranked subdivisions of genera.

***Ex. 1.*** *Agaricus* “tribus” [unranked] *Pholiota* Fr. (Syst. Mycol. 1: 240. 1821), sanctioned in the same work, is the validly published basionym of the generic name *Pholiota* (Fr.) P. Kumm. (Führer Pilzk.: 22. 1871) (see Art. 41 Ex. 9).

**ARTICLE F.5**

**REGISTRATION OF NAMES AND NOMENCLATURAL ACTS**

***F.5.1.*** In order to be validly published, nomenclatural novelties (Art. 6 Note 4) applied to organisms treated as fungi under this *Code* (Pre. 8; including fossil fungi and lichen-forming fungi) and published on or after 1 January 2013 must, in the protologue, include citation of the identifier issued for the name by a recognized repository (Art. F.5.3).

***Ex. 1.*** The protologue of *Albugo arenosa* Mirzaee & Thines (in Mycol. Prog. 12: 50. 2013) complies with Art. F.5.1 because it includes citation of “MB 564515”, an identifier issued by MycoBank, one of three recognized repositories. The decision by the Nomenclature Committee for Fungi to appoint (Art. F.5.3) Fungal Names, Index Fungorum, and MycoBank as repositories (Redhead & Norvell in Taxon 62: 173–174. 2013) was ratified (Art. F.5.3) by the 10th International Mycological Congress (May in Taxon 66: 484. 2017).

***Ex. 2.*** The designation “*Austropleospora archidendri*” (Ariyawansa & al. in Fungal Diversity 75: 64. 2015) is not a validly published new combination based on *Paraconiothyrium archidendri* Verkley & al. (in Persoonia 32: 37. 2014) because it was published without citing an identifier issued by a recognized repository, even though the recognized repository Index Fungorum had previously issued the identifier “IF 551419” for the intended new combination.

***Ex. 3.*** The designation *“Priceomyces fermenticarens*” (Gouliamova & al. in Persoonia 36: 429. 2016), intended as a new combination, was published with the identifier “MB 310255”, which refers to the identifier “IF 310255” that had been assigned to the intended basionym, *Candida fermenticarens* Van der Walt & P. B. Baker (in Bothalia 12: 561. 1978) by Index Fungorum prior to registration becoming mandatory. The recognized repository MycoBank assigned the identifier “MB 818676” for the intended new combination after its publication, but because no identifier was issued prior to its publication the intended combination was not validly published. *Priceomyces fermenticarens* (Van der Walt & P. B. Baker) Gouliam. & al. (in Persoonia 39: 289. 2017) was subsequently validly published with citation of the identifier “MB 818692”, newly issued by MycoBank.

***F.5.2.*** For an identifier to be issued by a recognized repository as required by Art. F.5.1, the minimum elements of information that must be accessioned by author(s) of scientific names are the proposed name itself and those elements required for valid publication under Art. 38.1(a) and 39.2 (validating description or diagnosis) and Art. 40.1 and 40.7 (type) or Art. 41.5 (reference to the basionym or replaced synonym). When the accessioned and subsequently published information for a name with a given identifier differ, the published information is considered definitive.^2^

***Note 1.*** Issuance of an identifier by a recognized repository presumes subsequent fulfilment of the requirements for valid publication of the name (Art. 32–45, F.5.1, and F.5.2) but does not in itself constitute or guarantee valid publication.

***Note 2*****.** The words “name” and “names” are used in Art. F.5.1 and F.5.2 for names that may not yet be validly published, in which case the definition in Art. 6.3 does not apply.

***F.5.3.*** The Nomenclature Committee for Fungi (see Div. III Prov. 7) has the power to *(a)* appoint one or more localized or decentralized, open and accessible electronic repositories to accession the information required by Art. F.5.2 and F.5.5 and issue the identifiers required by Art. F.5.1 and F.5.4; *(b)* cancel such appointment at its discretion; and *(c)* set aside the requirements of Art. F.5.1, F.5.2, F.5.4, and F.5.5, should the repository mechanism, or essential parts thereof, cease to function. Decisions made by this Committee under these powers are subject to ratification by a subsequent International Mycological Congress.

***F.5.4.*** For purposes of priority (Art. 9.19, 9.20, and 10.5), designation of a type, on or after 1 January 2019, of the name of an organism treated as a fungus under this *Code* (Pre. 8), is achieved only if an identifier issued for the type designation by a recognized repository (Art. F.5.3) is cited.

***Note 3.*** Art. F.5.4 applies only to the designation of lectotypes (and their equivalents under Art. 10), neotypes, and epitypes; it does not apply to the designation of a holotype when publishing the name of a new taxon, for which see Art. F.5.2.

***F.5.5.*** For an identifier to be issued by a recognized repository as required by Art. F.5.4, the minimum elements of information that must be accessioned by author(s) of type designations are the name being typified, the author designating the type, and those elements required by Art. 9.21, 9.22, and 9.23.

***Note 4.*** Issuance of an identifier by a recognized repository presumes subsequent fulfilment of the requirements for effective type designation (Art. 7.8–7.11 and F.5.4) but does not in itself constitute or guarantee a type designation.

***F.5.6.*** When the identifier issued for a name by a recognized repository is cited incorrectly in the protologue, this is treated as a correctable error not preventing valid publication of the name, provided that the identifier was issued prior to the protologue.

***Ex. 4.*** The identifier “MB 564220” was issued by MycoBank for *Cortinarius peristeris* Soop (in Bresadoliana 1: 22. 2013) prior to publication of the name. Even though the identifier was incorrectly cited as “MB 564” in the protologue, the name is validly published.

***F.5.7.*** An identifier remains associated with the name or designation for which it was issued. If, when published, a designation for which an identifier has been issued does not meet other requirements for valid publication, in order for that designation to become a validly published name, a new identifier must be obtained.

***Ex. 5.*** The designation *“Nigelia*” (Luangsa-ard & al. in Mycol. Progr. 16: 378. 2017) was published without citation of an identifier. MycoBank assigned the identifier “MB 823565” for this designation after publication. The designation was later validated as *Nigelia* Luangsa-ard & al. (in Index Fungorum 345: 1. 2017) with citation of the identifier “IF 553229” newly issued by Index Fungorum.

***F.5.8.*** When the identifier issued for a type designation by a recognized repository is cited incorrectly in the typifying publication, this is treated as a correctable error not preventing designation of the type, provided that the identifier was issued prior to the typifying publication.

**Recommendation F.5A**

***F.5A.1.*** Authors of names of organisms treated as fungi are encouraged to *(a)* deposit the required elements of information for any nomenclatural novelty in a recognized repository as soon as possible after a work is accepted for publication, so as to obtain identifiers for each nomenclatural novelty; *(b)* inform the recognized repository that issued the identifier of the complete bibliographic details upon publication of the name, including volume and part number, page number, date of publication, and (for books) the publisher and place of publication; and *(c)* upon publication of a name, supply an electronic version of the publication to the recognized repository that issued the identifier associated with the name.

***F.5A.2.*** In addition to meeting the requirements for effective publication of choices of name (Art. 11.5 and 53.5), orthography (Art. 61.3), or gender (Art. 62.3), those publishing such choices for names of organisms treated as fungi are encouraged to record the choice in a recognized repository (Art. F.5.3) and cite the identifier in the place of publication.

**SECTION 3**

**REJECTION OF NAMES**

**ARTICLE F.6**

***F.6.1.*** The name of a taxon treated as a fungus published on or after 1 January 2019 is illegitimate if it is a later homonym of a prokaryotic or protozoan name (see also Art. 54 and Rec. 54A).

**ARTICLE F.7**

***F.7.1.*** In the interest of nomenclatural stability, for organisms treated as fungi, lists of names proposed for rejection may be submitted to the General Committee, which will refer them to the Nomenclature Committee for Fungi (see Div. III Prov. 2.2, 7.9, and 7.10) for examination by subcommittees established by that Committee in consultation with the General Committee and appropriate international bodies. Names on these lists, which become part of the Appendices of the *Code* once reviewed and approved by the Nomenclature Committee for Fungi and the General Committee (see Art. 56.3 and Rec. 56A.1), are to be treated as rejected under Art. 56.1, except that they may become eligible for use by conservation under Art. 14 (see also Art. F.2.1).

**SECTION 4**

**NAMES OF FUNGI WITH A PLEOMORPHIC LIFE CYCLE**

**ARTICLE F.8**

***F.8.1.*** A name published prior to 1 January 2013 for a taxon of non-lichen-forming *Ascomycota* and *Basidiomycota,* with the intent or implied intent of applying to or being typified by one particular morph (e.g. anamorph or teleomorph; see Note 2), may be legitimate even if it otherwise would be illegitimate under Art. 52 on account of the protologue including a type (as defined in Art. 52.2) referable to a different morph. If the name is otherwise legitimate, it competes for priority (Art. 11.3 and 11.4).

***Ex. 1.*** *Penicillium brefeldianum* B. O. Dodge (in Mycologia 25: 92. 1933) was described and based on a type with both the anamorph and teleomorph (and therefore necessarily typified by the teleomorph element alone under editions of the *Code* prior to the *Melbourne Code* of 2012). The combination *Eupenicillium brefeldianum* (B. O. Dodge) Stolk & D. B. Scott (in Persoonia 4: 400. 1967) for the teleomorph is legitimate. *Penicillium dodgei* Pitt (Gen. Penicillium: 117. 1980), typified by the anamorph in a dried culture “derived from Dodge’s type”, did not include the teleomorphic type of *P. brefeldianum* and therefore it too is legitimate. However, when considered a species of *Penicillium,* the correct name for all its states is *P. brefeldianum*.

***Note 1.*** Except as provided in Art. F.8.1, names of fungi with mitotic asexual morphs (anamorphs) as well as a meiotic sexual morph (teleomorph) must conform to the same provisions of this *Code* as all other fungi.

***Note 2.*** Editions of the *Code* prior to the *Melbourne Code* of 2012 provided for separate names for mitotic asexual morphs (anamorphs) of certain pleomorphic fungi and required that the name applicable to the whole fungus be typified by a meiotic sexual morph (teleomorph). Under the current *Code,* however, all legitimate fungal names are treated equally for the purposes of establishing priority, regardless of the life-history stage of the type (see also Art. F.2.1).

***Ex. 2.*** *Mycosphaerella aleuritidis* (Miyake) S. H. Ou (in Sinensia 11: 183. 1940), when published as a new combination, was accompanied by a Latin diagnosis of the newly discovered teleomorph corresponding to the anamorph on which the basionym *Cercospora aleuritidis* Miyake (in Bot. Mag. (Tokyo) 26: 66. 1912) was typified. Under editions of the *Code* prior to the *Melbourne Code* of 2012, *M. aleuritidis* was considered to be the name of a new species with a teleomorph type, dating from 1940, and with authorship attributed solely to Ou. Under the current *Code,* the name is cited as originally published, *M. aleuritidis* (Miyake) S. H. Ou, and is typified by the type of the basionym.

***Ex. 3.*** In the protologue of the teleomorph-typified *Venturia acerina* Plakidas ex M. E. Barr (in Canad. J. Bot. 46: 814. 1968) the anamorph-typified *Cladosporium humile* Davis (in Trans. Wisconsin Acad. Sci. 19: 702. 1919) was included as a synonym. Because it was published prior to 1 January 2013, the name *V. acerina* is not illegitimate, but *C. humile* is the earliest legitimate name at the rank of species.

***Note 3.*** Names proposed simultaneously for separate morphs (e.g. anamorph and teleomorph) of a taxon of non-lichen-forming *Ascomycota* and *Basidiomycota* are necessarily heterotypic and are not therefore alternative names as defined by Art. 36.3.

***Ex. 4.*** *Hypocrea dorotheae* Samuels & Dodd and *Trichoderma dorotheae* Samuels & Dodd were simultaneously validly published (in Stud. Mycol. 56: 112. 2006) for what the authors considered a single species with *Samuels & Dodd 8657* (PDD 83839) as the holotype. Because these names were published before 1 January 2013 (see Art. F.8.1 and Note 2), and because the authors explicitly indicated that the name *T. dorotheae* was typified by the anamorphic element of PDD 83839, both names are validly published and legitimate. They are not alternative names as defined in Art. 36.3.

**SECTION 5**

**ORTHOGRAPHY OF NAMES**

**ARTICLE F.9**

***F.9.1.*** Epithets of fungal names derived from the generic name of an associated organism are to be spelled in accordance with the accepted spelling of the name of that organism; other spellings are regarded as orthographical variants to be corrected (see Art. 61).

***Ex. 1.*** *Phyllachora ‘anonicola’* Chardón (in Mycologia 32: 190. 1940) is to be corrected to *P. annonicola* in accordance with the accepted spelling of *Annona* L.; *Meliola ‘albizziae’* Hansf. & Deighton (in Mycol. Pap. 23: 26. 1948) is to be corrected to *M. albiziae* in accordance with the accepted spelling of *Albizia* Durazz.

***Ex. 2.*** *Dimeromyces ‘corynitis’* Thaxter (in Proc. Amer. Acad. Arts 48: 157. 1912) was stated to occur “On the elytra of *Corynites ruficollis* Fabr.”, but the name of the host, a species of beetle, is correctly spelled *Corynetes ruficollis*. The fungal name is therefore to be spelled *D. corynetis*.

**SECTION 6**

**AUTHOR CITATIONS**

**ARTICLE F.10**

***F.10.1.*** For names of organisms treated as fungi, the identifier issued for the name by a recognized repository (Art. F.5.1) may be used subsequent to the protologue in place of an author citation for the name but not to replace the name itself (see also Art. 22.1 and 26.1).

**Recommendation F.10A**

***F.10A.1.*** An identifier used in place of an author citation as permitted by Art. F.10.1 should be presented with the symbol # preceding the numerical part of the identifier, and the resulting string should be enclosed in square brackets. In electronic publications, this string should be provided with a direct and stable link to the corresponding record in one of the recognized repositories.

***Ex. 1.*** *Astrothelium meristosporoides* [#816706]. The direct and stable link to a record in a recognized repository would be either http://www.mycobank.org/MB/816706 or http://www.indexfungorum.org/Names/NamesRecord.asp?RecordID=816706.

## Abbreviations

Art.: Article (of the *Code*)
*Code*: *International Code of Nomenclature for algae, fungi, and plants*
Div: Division (of the *Code*)
FNS: Fungal Nomenclature Session
IBC: International Botanical Congress
IMC: International Mycological Congress
NCF: Nomenclature Committee for Fungi
Prov.: Provision (of Div. III of the *Code*)
Rec.: Recommendation (of the *Code*)
